# The complete chloroplast genome of *Casuarina cunninghamiana* (Casuarinaceae)

**DOI:** 10.1080/23802359.2021.2005492

**Published:** 2021-11-30

**Authors:** Zhen Li, Yong Zhang, Yong-cheng Wei, Jing-xiang Meng, Yu-jiao Wang

**Affiliations:** Research Institute of Tropical Forestry, Chinese Academy of Forestry, Guangzhou, P.R. China

**Keywords:** *Casuarina cunninghamiana*, complete chloroplast genome, phylogenetic analysis

## Abstract

*Casuarina cunninghamiana* Miq. naturally occurs in eastern Australia from New South Wales to north Queensland. After being introduced to China, it has become an important tree species of ecological shelter plantations in coastal areas of southern China. In this study, the complete chloroplast (cp) genome of *C. cunninghamiana* was sequenced and analyzed based on the Illumina NovaSeq 6000 platform. The cp genome of *C. cunninghamiana* was found to be 15,6129 bp in length, including a large single copy (LSC) region of 86,200 bp and a small single copy (SSC) region of 18,457 bp, which were separated by two inverted repeats (IRs) of 25,736 bp. The cp genome contains 132 genes, consisting of 87 protein-coding genes, 37 tRNA genes, and eight rRNA genes. The overall GC content of the cp genome was 36.34%. The phylogenetic analyses indicated that *C. cunninghamiana* was closely related to *C. glauca* and *C. equisetifolia* and clustered with 4 Betulaceae species.

*Casuarina cunninghamiana* Miq. is an important tree species with considerable ecological and economic values (Doran and Hall 1983; Jiang et al. 2012), which has been extensively planted as timber forest, windbreaks in the inland and coastal regions of China. It is one of the most successful tree species of Casuarinaceae introduced to China (Zhong and Bai 1996; Zhong et al. 2010). The phylogenetic relationship of Casuarinaceae with other plants is still controversial up to now (Beadle 1981; John and Wilson 1989). The chloroplast genome is a very effective tool for tracing the origin of species and migration, and has been widely used in phylogenetic analysis (Mehmood et al. 2020a, 2020b, 2020c). In this study, we obtained the complete chloroplast genome sequence of *C. cunninghamiana* and analyzed its phylogenetic relationships with other related species, which will help us clarify its phylogenetic status and contribute for further effective utilization.

In this study, the samples of *C. cunninghamiana* were collected from Raoping, Guangdong Province of China (23°35′15″N, 117°8′10″E). Total genomic DNA was extracted from young and fresh branchlets using E.Z.N.A^®^ Plant DNA kits (Omega Bio-Tek Inc., Norcross, GA, USA). A specimen was deposited at the Key Laboratory of National Forestry and Grassland Administration on Tropical Forestry Research, Research Institute of Tropical Forestry, Chinese Academy of Forestry (Zhen Li, lzlizhenlz@yeah.net, Guangzhou, China) under the voucher number RCCZCCRPLZ202009. A paired-end library with an insert size of 450 bp was constructed and the library was sequenced on the Illumina NovaSeq 6000 platform (BIOZERON Co., Ltd, Shanghai, China). Approximately 5.01 Gb of raw data from *C. cunninghamiana* were generated with 150 bp paired-end read lengths. The cp genome sequence of *C. cunninghamiana* was assembled using the program NOVOPlasty v4.2 with a 39-mer length and the genome range was from 120 kb to 200 kb (Dierckxsens et al. 2017). Then we used the software GeSeq to annotate chloroplast protein and rRNA-coding genes by BLAST with profile HMM hits based on protein search identity was set to 60 and rRNA, tRNA, DNA search identity was set to 35 (Tillich et al. 2017). The software tRNAscan-SE v2.0.7 was used to verify the tRNA genes with default settings (Lowe and Chan 2016). To obtain a high accurate gene set, we manually corrected the exon/intron boundaries and the head and tail of genes based on the reference genome (*Ostrya japonica* MG386375). Finally, we obtained the annotated complete chloroplast genome of *C. cunninghamiana* and submitted to GenBank with accession number MZ474954.

The complete chloroplast genome of *C. cunninghamiana* showed a typical quadripartite structure with the length of 15,6129 bp, including a large single copy (LSC) region of 86,200 bp, a small single copy (SSC) region of 18,457 bp, and two inverted repeat regions (IRs) of 25,736 bp. The base composition of the complete chloroplast genome existed differences, and the content of A, T, C, and G were 31.37%, 32.30%, 18.50%, and 17.83%, respectively. The overall GC content of the cp genome was 36.34%, and the corresponding values of the LSC, SSC, and IR regions were 34.16%, 29.72%, and 42.36%, respectively. A total of 132 genes were annotated, including 87 protein-coding genes, 37 tRNA genes, and eight rRNA genes. Most of them were in a single copy, while 19 genes (eight of the protein-coding genes, four rRNA genes, and seven tRNA genes) were duplicated in the IR regions.

To reveal the phylogenetic status of *C. cunninghamiana*, as well as Casuarinaceae, we reconstructed the phylogenetic relationships based on the cp genomes of three species from genus *Casuarina* and fifteen cp genomes from other species in Fagales. *Rosa praelucens* was served as outgroup. Genome sequences were downloaded from NCBI GenBank and were aligned by software MAFFT (Katoh and Standley 2013). The maximum likelihood bootstrap analyses with 1000 replicates were performed using MEGA (Kumar et al. 2016). As shown in the phylogenetic tree (Figure 1), *C. cunninghamiana* was closely related to *C. glauca* and *C. equisetifolia* and clustered with 4 Betulaceae species. In summary, this study provided essential data for phylogenetic and evolutionary analyses of *C. cunninghamiana*, as well as Casuarinaceae and will be useful in studies on its population genetics, molecular-assisted breeding, genetic resources evaluation and utilization.

**Figure 1.**
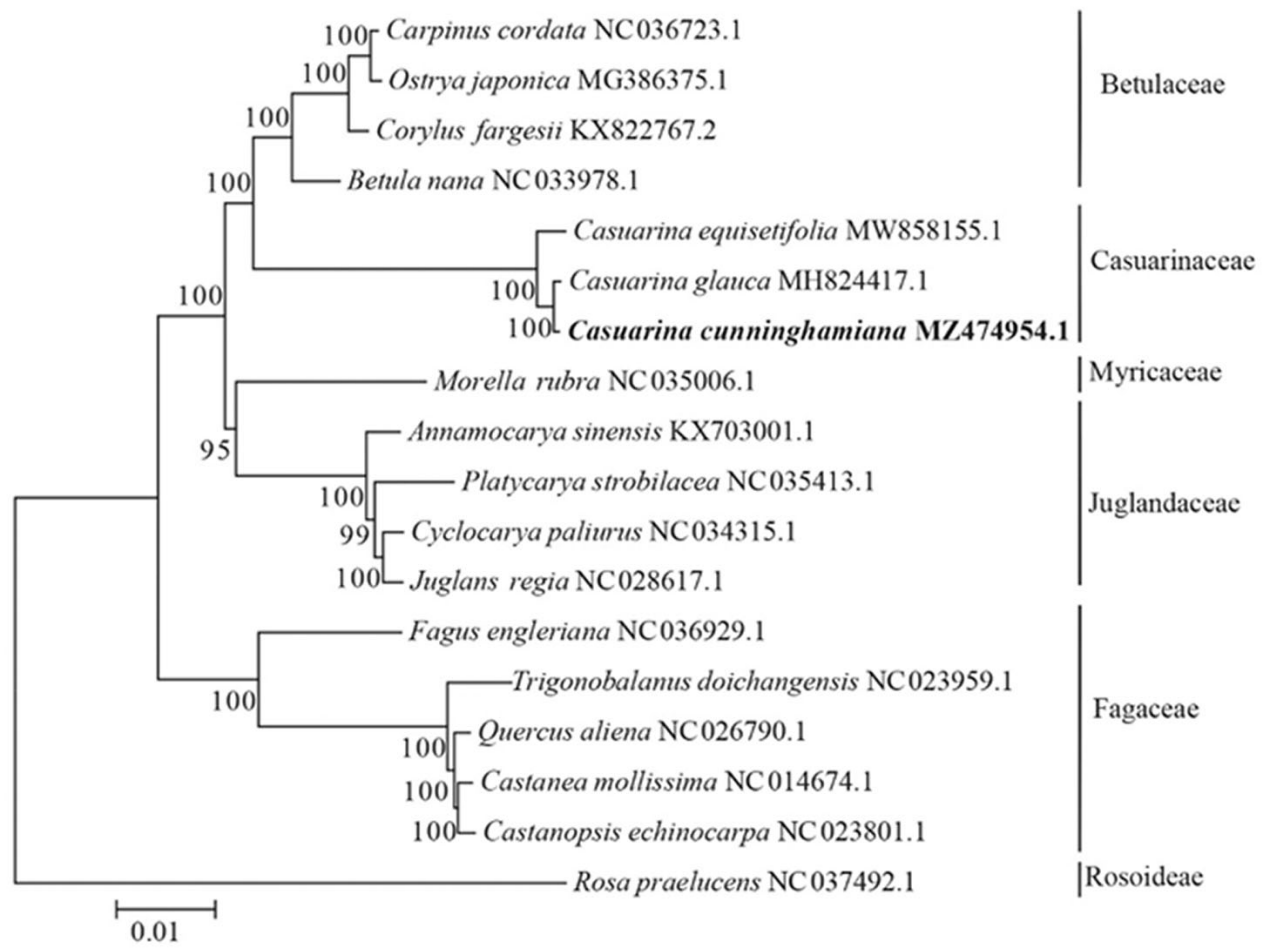
The phylogenetic tree was consturcted based on complete chloroplast genomes of 17 species from Fagales and one species from Rosoideae using Maximum-likelihood method. Numbers in the nodes are the bootstrap values from 1000 replicates.

## Data Availability

The genome sequence data that support the findings of this study are openly available in GenBank of NCBI under the accession No. MZ474954 at (https://www.ncbi.nlm.nih.gov/nuccore/mz474954). The associated BioProject, SRA and Bio-Sample numbers are PRJNA760639, SRR15729584 and SAMN21219931, respectively.

## References

[CIT0001] Beadle NCW. 1981. The vegetation of Australia. Cambridge: Cambridge university press.

[CIT0002] Dierckxsens N, Mardulyn P, Smits G. 2017. NOVOPlasty: de novo assembly of organelle genomes from whole genome data. Nucleic Acids Res. 45(4):e18. 28204566 10.1093/nar/gkw955 PMC5389512

[CIT0003] Doran JC, Hall N. 1983. Notes on fifteen Australian Casuarina species. In: Midgley SJ, Turnbull JW, Johnston RD, editors. Casuarina ecology. Management and utilization. Canberra: CSIRO, p. 19–52.

[CIT0004] Jiang QB, Zhang Y, Zhong CL, Zeng BS, Bogusz D, Franche C. 2012. Establishment of an in vitro plant regeneration protocol for *Casuarina cunninghamiana* Miq. via indirect organogenesis. New For. 43(2):143–154.

[CIT0005] John LAS, Wilson KL. 1989. Casuarinaceae: a sysnopsis. In: Carne PR, Blackmore S, editors. Evaluation, systematics and fossil history of the Hamamelide. Vol. 2: higher Hamamelide, systematics association special volume, No. 40B. Oxford: Clarendom Press, p. 167–188.

[CIT0006] Katoh K, Standley DM. 2013. MAFFT multiple sequence alignment software version 7: improvements in performance and usability. Mol Biol Evol. 30(4):772–780. 23329690 10.1093/molbev/mst010 PMC3603318

[CIT0007] Kumar S, Stecher G, Tamura K. 2016. MEGA7: molecular evolutionary genetics analysis version 7.0 for bigger datasets. Mol Biol Evol. 33(7):1870–1874. 27004904 10.1093/molbev/msw054 PMC8210823

[CIT0008] Lowe TM, Chan PP. 2016. tRNAscan-SE On-line: integrating search and context for analysis of transfer RNA genes. Nucleic Acids Res. 44(W1):W54–W57. 27174935 10.1093/nar/gkw413 PMC4987944

[CIT0009] Mehmood F, Abdullah Shahzadi I, Ahmed I, Waheed MT, Mirza B. 2020b. Characterization of *Withania somnifera* chloroplast genome and its comparison with other selected species of Solanaceae. Genomics. 112(2):1522–1530. 31470082 10.1016/j.ygeno.2019.08.024

[CIT0010] Mehmood F, Abdullah Ubaid Z, Bao YM, Poczai P, Mirza B. 2020c. Comparative Plastomics of Ashwagandha (*Withania*, Solanaceae) and Identification of Mutational Hotspots for Barcoding Medicinal Plants. Plants. 9(6):752. 10.3390/plants9060752 PMC7355740 32549379

[CIT0011] Mehmood F, Abdullah Ubaid Z, Shahzadi I, Ahmed I, Waheed MT, Poczai P, Mirza B. 2020a. Plastid genomics of Nicotiana (Solanaceae): insights into molecular evolution, positive selection and the origin of the maternal genome of Aztec tobacco (*Nicotiana rustica*). PeerJ. 8:e9552. 32775052 10.7717/peerj.9552 PMC7382938

[CIT0012] Tillich M, Lehwark P, Pellizzer T, Ulbricht-Jones ES, Fischer A, Bock R, Greiner S. 2017. GeSeq - versatile and accurate annotation of organelle genomes . Nucleic Acids Res. 45(W1):W6–W11. 28486635 10.1093/nar/gkx391 PMC5570176

[CIT0013] Zhong CL, Bai JY. 1996. Introduction trials of Casuarinas in southern China. In: Pinyopusarerk K, Turnbull JW, Midgley SJ, editors. Recent Casuarina research and development. Canberra: CSIRO, p. 191–195.

[CIT0014] Zhong CL, Zhang Y, Chen Y, Jiang QB, Chen Z, Liang JF, Pinyopusarerk K, Franche C, Bogusz D. 2010. Casuarina research and applications in China. Symbiosis. 50(1–2):107–114.

